# Topics of the Month

**Published:** 1892-10

**Authors:** 


### TOPICS OF THE MONTH.

If, in spite of all reasonable precautions, cholera should gain a foothold in this country, the Hamburg-American Packet Steamship Company will have a fearful reckoning to make with the people. There would seem to be no excuse for such conduct as has been manifest on the part of this steamship company. In the face of the fact that the line sails from an infected seaport, where hundreds of deaths of cholera have been taking place daily, this line has brought hundreds, even thousands, of passengers to our shores, where nothing but the strictest quarantine has prevented their carrying this disease to the remote parts of the United States. There would seem to be reason for an adequate law to prevent a repetition of such conduct in the future.

---

In the light of recent events, the argument in favor of a National quarantine in all the important seaports is strengthened. The quibbling over jurisdiction will then be stopped. We shall hear no more of injunctions to restrain the health officer from performing his duty. Passengers seem to be the most unreasonable people in the world. In one moment they beseech the health officer to put them ashore, and as soon as he can get adequate authority to do so, he is resisted by the residents from landing passengers; whereupon the passengers turn upon the health officer and curse him for all their woes. This happens in spite of the fact that the health officer is straining every nerve to serve the best interests of all the people. It is true that he is hampered on the one side by the very fact that he is a State official, appointed through political influence, and, on the other hand, by the fact that he has a most inordinate egotism; but this has not prevented him from attempting to serve the very passengers who now condemn him. Let us have a National quarantine, and all this petty quarrel will end.

---

Col. Weber, the United States Commissioner of Immigration, seems to think that there is an unnecessary alarm among the people with reference to cholera. It is all very well for a non-scientific official to speak in this assuring manner, but scientists know the history of the progress of epidemics. The germ once planted will grow its deadly fruit. It is carried westward by the tide of immigration, and, unless stopped by some almost miraculous display of human judgment, will continue to travel until it expends its forces by the stamping-out process of germ-destroying agents. Let no one feel too sure. These immigrants bring the germ in their clothing and on their persons, and in various ways it lurks around and about their bodies for a long period. The frost of Winter may serve to stay its ravishing deadliness, but with the warmth of Spring and Summer it may be revived with all its direful force. To be forewarned is to be forearmed ; the better way would be to prevent a single immigrant from an infected or suspicious port from setting foot on our shores for a year.

---

The promptitude with which the Governor of the State of New York responded to the demands made for active support is commendable. The only wonder is that the National Guard was not placed on duty at once at the moment of the purchase of the property at Fire Island, and a detachment of sheriff's officers, specially sworn, should have been placed on duty. The purchase, too, should have been kept a secret until the property was securely occupied by the State authorities. It is very easy to say what should have been done in the light of after events. Let us take warning of the experience of the present, and provide against such humiliating complications in future.

---

The *Charlotte Medical Journal*, edited by Drs. E. C. Register and J. C. Montgomery, is a handsome addition to the periodical literature of the day. The size of the page is a little greater than the ordinary journals, being large octavo, but its type is proportionately larger, and is very pleasant to the eye. We hope our North Carolina contemporary will find ample support and remain with us during all time.

---

Dr. Joseph Price, of Philadelphia, has offered a prize of $100 to be awarded to the Fellow of the Medical Society of Virginia, who may present, during the session of 1893, the best essay on the history of surgery and surgeons of Virginia. Dr. Price is a native of Virginia, and thus pays a fitting compliment to the State of his birth. The rules governing the award of the prize are to be in all respects similar to those relating to the Hunter McGuire annual prize.

---

The *Brooklyn Medical Journal*, with commendable enterprise, published a special edition, under date of September 10, 1892, devoted entirely to the consideration of cholera. It is an official report of the proceedings of a special meeting of the Medical Society of the County of Kings, held September 6, 1892, and contains the following papers : Work in the Department of Health in

Anticipation of Cholera, and Plans for its Management in the Event of its Introduction," by John Griffin, M. D., Commissioner of Health; Bacteriology of Cholera, and Methods of Disinfection, by George M. Sternberg, M. D., Deputy Surgeon-General U. S. Army; The Hygiene of Cholera, by Albert L. Gihon, M. D., Medical Director U. S. Navy, Ex-President American Public Health Association; Practical Sanitation vs. Detention, by A. N. Bell, M. D., editor of the *Sanitarian*; Personal Experience with Cholera in China and Japan, by E. S. Bogart, M. D., Medical Director U. S. Navy; Practical Suggestions for the Management of Cholera, by Charles V. Gavatt, Surgeon U. S. Navy; Cholera Epidemic of 1848 and 1854 in Brooklyn, by Andrew Otterson, M. D., Ex-Commissioner of Health; Cholera Epidemic of 1866 in Brooklyn, by F. H. Colton, Sanitary Inspector of the Metropolitan Board of Health; The Preparation in the City of Brooklyn for the Anticipated Epidemic of Cholera of 1884-1885, by Joseph H. Raymond, M. D., Ex-Commissioner of Health; The Treatment of Cholera, by John A. McCorkle, Professor of the Principles and Practice of Medicine, Long Island College Hospital; A Typhoid Case of Cholera, by S. E. Fuller, M. D.; Pathological Findings in Cholera, by Joshua M. VanCott, Jr., M. D., Professor of Histology and Pathological Anatomy, Long Island College Hospital. This valuable group of papers ought to be carefully read by every physician, and especially by health officers and sanitarians. We have no doubt that copies can be obtained upon application to the business manager of the *Journal*, 333 Hancock street, Brooklyn, N. Y.

---

THE medical colleges of Buffalo were both opened on Monday, September 26th, for the sessions of 1892-93. The Buffalo University began its term in the old college building, but it expects to have the new one in readiness for occupation during the Christmas season. The Niagara University starts with a promising increase in attendance, and renewed energy in its teaching faculty. The influence of the State Medical Examining and Licensing Board is to stimulate a greater thoroughness on the part of the medical colleges throughout the State with reference to the quality of their teaching, as well as to a surveillance over the equipment of their students at the entrance examination. This is as it should be, and serves to bring down the rejections by the State Board to the minimum.

THE *Hot Springs Medical Monthly* deservedly roasts the *Doctors' Weekly* for purloining its paragraphs without credit. We have observed this practice on the part of the *Doctors' Weekly* for some time, and we are glad our Hot Springs contemporary has called down the King.

